# An open-source multi-semantic annotation dataset and automated recognition tool for viral carcinogenesis factors

**DOI:** 10.1093/database/baaf038

**Published:** 2025-09-24

**Authors:** Honglian Huang, Danqi Huang, Ziyi Wei, Yanling Qi, M James C Crabbe, Xiaoyan Zhang, Ying Wang

**Affiliations:** Department of Bioinformatics, School of Life Sciences and Technology, Tongji University, 1239 Siping Road, Shanghai, 200092, China; Department of Clinical Laboratory Medicine Center, Yueyang Hospital of Integrated Traditional Chinese and Western Medicine, Shanghai University of Traditional Chinese Medicine, 110 Ganhe Road, Shanghai, 200437, China; Department of Bioinformatics, School of Life Sciences and Technology, Tongji University, 1239 Siping Road, Shanghai, 200092, China; Department of Bioinformatics, School of Life Sciences and Technology, Tongji University, 1239 Siping Road, Shanghai, 200092, China; Department of Electronic Information, School of Health Science and Engineering, University of Shanghai for Science and Technology, 516 Jungong Road, Shanghai, 200093, China; Wolfson College, Oxford University, Linton Road, Oxford, OX2 6UD, United Kingdom; Institute of Biomedical and Environmental Science & Technology, University of Bedfordshire, University Square, Luton, LU1 3JU, United Kingdom; School of Life Sciences, Shanxi University, 92 Basin City Road, Taiyuan, 030006, China; Department of Bioinformatics, School of Life Sciences and Technology, Tongji University, 1239 Siping Road, Shanghai, 200092, China; Department of Bioinformatics, School of Life Sciences and Technology, Tongji University, 1239 Siping Road, Shanghai, 200092, China; Department of Clinical Laboratory Medicine Center, Yueyang Hospital of Integrated Traditional Chinese and Western Medicine, Shanghai University of Traditional Chinese Medicine, 110 Ganhe Road, Shanghai, 200437, China

## Abstract

In-depth investigations into the characteristics of high-risk oncogenic viruses are critical for the early prevention and control of related cancers and the development of effective vaccines. The mechanism of viral carcinogenesis involves numerous risk factors such as viral genomic variations, lifestyle, and environmental influences. Based on literature data on eight oncogenic viruses, we have created a large-scale, semantically rich corpus of viral carcinogenic factors, including 551 715 abstracts and 5 821 308 entities, using natural language processing technology combined with expert knowledge. We also developed a semantic filter to improve entity recognition performance. Moreover, transcriptomic data related to oncogenic viruses were collected. We performed gene differential expression analysis, feature gene identification, and immune microenvironment analysis. A visual knowledge platform, an open-source dataset, and a tool for automatically identifying internal and external semantic factors related to viral carcinogenesis are available at http://www.biomedinfo.cn:8281/. This study provides new insights into the key factors involved in the viral carcinogenesis process and helps researchers and clinicians quickly obtain clues for further experimental research and clinical validation.

## Introduction

Viral infections are significant factors contributing to tumour susceptibility. The International Agency for Research on Cancer has classified the following potentially carcinogenic viruses [1]: human papilloma virus (HPV) [2], human immunodeficiency virus (HIV) [3], Epstein–Barr virus (EBV) [4], Merkel cell polyomavirus (MCV) [5], hepatitis C virus (HCV) [6], human T-cell leukemia virus type 1 (HTLV-1) [7], hepatitis B virus (HBV) [8], and Kaposi’s sarcoma herpesvirus (KSHV) [9]. Factors linking oncogenic viruses and cancer development can be categorized into internal and external factors. Internal factors can include genetic, immune, or gene functions [10]. For example, viruses can integrate into the host genome, resulting in mutations in host genes and increased genomic instability, which ultimately leads to a higher risk of cancer [11]. External factors include lifestyle, environment, and geographic location. For instance, HTLV-1 infection shows geographic variability, commonly occurring in Japan and Africa [12]. Therefore, analysing the relationship between oncogenic viruses and cancer from both internal and external perspectives can aid in the prevention and treatment of cancers associated with these viruses at multiple levels.

Currently, research on key factors in virus-induced cancer development has been approached through three main aspects: (i) focusing on a single virus as research subject, e.g. HBV, which has been widely confirmed to be closely associated with hepatocellular carcinoma [13]; (ii) collecting specific types of data for analysis, such as host lifestyles and environmental exposure. Studies have shown that HPV and cigarette smoke condensate in lung cells suggest cooperation during carcinogenesis [14]; and (iii) utilizing omics data to investigate the molecular mechanisms and pathological impact of oncogenic viral infections on hosts. For example, interactions among the nasopharyngeal carcinoma tumour microenvironment, EBV infection, and tumour cells have been investigated using single-cell sequencing [15]. The recent application of multi-omics technologies has provided important insights into how viruses regulate host gene expression and signalling pathways. However, these methods are limited by a narrow data scope, struggle to provide comprehensive comparisons between different oncogenic viruses, and provide inconsistent analytical results among laboratories. Furthermore, research findings can be dispersed and fragmented.

Text- and data-mining techniques have been applied to address these issues. The rapid expansion of biomedical literature and sequencing data now offers a wealth of information about biomedical entities, including lifestyle factors, behavioural habits, genes, etc. Acquiring such knowledge can support studies on viruses and cancer development. For example, meta-analyses have shown that patients with chronic HCV infection have not only an increased incidence of hepatocellular carcinoma but also increased risk of intrahepatic cholangiocarcinoma, pancreatic cancer, and non-Hodgkin lymphoma [16]. Hepatocellular carcinoma associated with HBV in infected individuals is related to not only the immune system, but also lifestyles such as alcohol consumption, which can exacerbate liver damage. The risk of liver cancer is higher in patients infected with HBV who consume alcohol than in those with only HBV infection or those who consume alcohol without the virus [16, 17].

However, extracting and integrating the key factors of viral carcinogenesis from the literature still poses several challenges. Although the advent of named entity recognition (NER) tools has addressed issues such as the reliance on expert prior knowledge for manual annotation, slow annotation speed, low efficiency, and high costs, NER tools can currently only accurately annotate entities limited to a few common biomedical semantics, such as diseases, genes, compounds, and species. Semantic types related to lifestyle and dietary habits either are not covered or have poor recognition performance. Conversely, the development of NER tools for these semantic types is also constrained by the availability of existing expert-annotated datasets. Moreover, the accuracy of semantic recognition and normalization remains insufficient and requires further improvement. To improve NER tools and provide a more comprehensive coverage of biomedical concepts related to viral carcinogenic key factors, our goal was to extract various types of viral carcinogenic key factors from a vast amount of biomedical literature. We aimed to develop a semantically rich, large-scale viral annotation corpus and create a semantic filter to improve the accuracy of entity recognition. We also integrated omics data and included eight cancer-associated viruses for visualization. The study can assist research and clinical investigators to rapidly identify new leads for further exploration and clinical validation, thus providing new insights into the roles of key viral features in carcinogenesis.

## Materials and methods

### Data source

We obtained standardized terms and synonyms from the Medical Subject Headings database for each virus. We then applied them to search relevant PubMed abstracts. Supplementary Table S1 shows specific search terms for each virus.

### Ontology/semantic type selection and recognition

The Unified Medical Language System (UMLS) is a comprehensive biomedical vocabulary that includes over 3.3 million biomedical concepts from >200 source vocabularies and defines 127 semantic types [18]. We selected 43 from 127 semantic types related to viral carcinogenesis and divided them into 38 external and 5 internal factors based on their biological significance (Supplementary Table S2). External semantics refer to macroscopic factors in the relationship between oncogenic viral infections and cancer, such as the impact of the living environment on viral infection processes and cancer development. Internal semantics refer to microscopic factors, such as changes in gene function during viral infection and cancer progression. The UMLS concepts in the text were recognized and normalized using MetaMap 2020.

### Semantic filter construction

**Figure 1.**
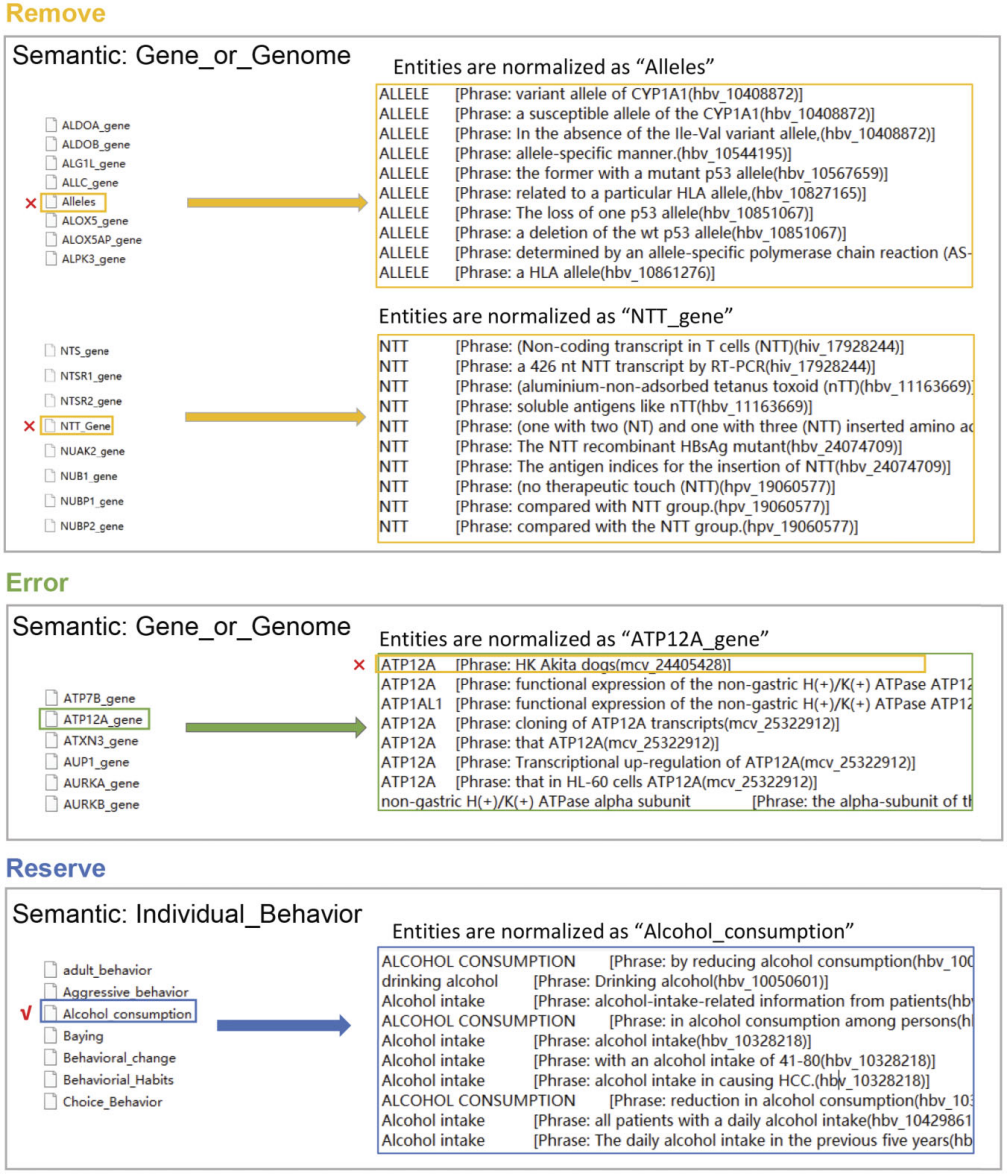
Semantic filters for Remove, Error, and Reserve. On the left are normalized terms, and on the right are UMLS concepts recognized by MetaMap from the original phrases. These UMLS concepts are normalized to the term on the left.

The MetaMap process of recognizing and normalizing biomedical entities still produces some false positives. MetaMap uses a dictionary-based approach to match entities in the text with dictionary concepts. This process typically leads to two types of errors: the first type occurs when all entities matched to a particular dictionary concept are incorrect; the second type occurs when only some of the entities matched to a concept are incorrect, while the rest are correct. These errors arise primarily due to ambiguity between UMLS concepts and entities where the meanings they refer to differ, leading to discrepancies in entity recognition. To improve accuracy, the results from MetaMap were manually reviewed and annotated. During this process, we summarized and categorized the results to construct three types of semantic filters: Remove, Error, and Reserve (Fig. 1).

To address the first type of error, we used the Remove or Reserve filter. The Remove filter was used to exclude incorrectly identified entities or those lacking biological significance, while the Reserve filter was used to retain correctly recognized entities that have biological significance. To optimize computational efficiency and reduce processing time, the program set an appropriate filter based on the ratio of correctly to incorrectly identified entities for each semantic type. For example, in the semantic type ‘Gene_or_Genome’, the percentage of entity misidentification cases was lower. Therefore, the Remove filter was applied to eliminate entities recognized and normalized as biologically meaningless (e.g. ‘Allele’) or those that were completely misidentified and incorrectly normalized (e.g. ‘NTT_gene’). In contrast, most entities classified as ‘Individual_Behaviour’ are too general to have specific biological significance, with only a few being correctly recognized and biologically meaningful. Therefore, the Reserve filter was applied to retain correctly recognized entities that have biological significance (e.g. ‘Alcohol consumption’).

For the second type of error, we used the Error module. The Error filter was used to remove incorrectly recognized entities. For example, MetaMap mistakenly normalized ‘HK’ in ‘HK Akita dogs’ to ‘ATP12A gene’. While ‘ATP12A gene’ is a valid biological term, ‘HK’ in this context actually stands for ‘high potassium’ and does not refer to a gene. This error occured because ‘ATP12A gene’ has a synonym that includes ‘HK’. As a result, ‘HK’ was excluded from being incorrectly normalized as ‘ATP12A gene’.

### Evaluation of semantic filters

We compared the entity recognition performance before and after applying the filters using the MedMention test set to evaluate the performance of the semantic filters. MedMention is a large-scale UMLS concept biomedical annotation dataset that includes 4392 PubMed abstracts, with 879 abstracts designated as the test set [19]. We further excluded entities with overly broad biological meanings, such as ‘Genes’ and ‘Wild Type’. We then evaluated the performance of MetaMap with and without the filters.

### Analysing the importance of carcinogenic factors

We analysed the importance of the carcinogenic factors by applying methods based on word counts, term frequency–inverse document frequency (TF–IDF), and term co-occurrence. We allocated the eight viruses to 43 semantic corpora for TF–IDF calculation. The TF–IDF value for each semantic corpus was computed, and the values for the entities were summed to assess their importance. We then calculated the co-occurrence frequency of viral terms and semantic types in the literature, meaning that the co-occurrence count increased by one every time a viral term and semantic type appeared together in the same abstract. The Fisher test was used to calculate the *P*-value for co-occurrence, and the log(*P*-value) was taken to represent the degree of co-occurrence between the virus terms and the semantic type.

### Omics data analysis

Sequencing data related to virus-associated diseases were collected from the GEO database. We removed batch effects among samples by processing the data using the removeBatchEffect() function in the limma [20] package. We then analysed differentially expressed genes (DEGs) in the processed expression data using the GEO2R [21] tool. The main R packages used in the analysis were Biobase [22] and GEOquery (version 2.40.0) [23]. We identified feature genes between samples that were infected or not with a virus using logistic regression analysis of DEGs. Furthermore, we used xCell [24] to analyse cell infiltration within the immune microenvironment and explored correlations between feature genes and immune cell infiltration.

### Platform development

We used the Django tool to visualize the text and omics data to better present the text- and data-mining results for different viruses and improve the functionality and readability of the results.

## Results

### Data source

A total of 551 715 abstracts related to cancer-associated viruses were collected, with HIV literature comprising the largest proportion at 302 060 articles. Using MetaMap combined with manual annotation, we annotated a total of 5 821 308 entities and 32 027 unique entities from these documents. Table 1 shows the number of documents, entities, and unique entities according to virus type.

**Table 1.** Number of virus literatures and entities.

Virus	PMID	Entities	Unique entities
MCV	6088	61 281	4874
HBV	55 478	620 215	10 718
HIV	302 060	2 878 912	24 187
HTLV1	11 572	137 705	5323
KSHV	19 623	268 441	8998
HPV	49 960	590 082	10 963
HCV	68 278	796 132	11 164
EBV	38 657	468 545	12 425
Total	551 715	5 821 308	32 027

### Ontology/semantic type selection and recognition

The virus annotation dataset defines these entities into 43 semantic types, divided into 38 external semantics and 5 internal semantics. The internal semantics include ‘Gene_or_Genome’, ‘Genetic_Function’, ‘Immunologic_Factor’, ‘Amino_Acid_Sequence’, and ‘Biologic_Function’, which are related to molecular mechanisms and functional changes during viral or virus–host cancer development. The 38 external semantics primarily include risk factors for changes in the organism caused by external hazardous environments. Supplementary Table S3 shows the number of entities in each internal and external semantic type for each virus. The most prevalent semantic type was ‘Disease_or_Syndrome’, accounting for 30.3% of all named entities in the dataset followed by ‘Virus’, with 19.6% of the entities annotated as this type. The less frequent semantic types were ‘Animal’, ‘Activity’, and ‘Environmental_Effect_of_Humans’. In addition to entity recognition and classification, we also normalized the entities by mapping them to biomedical concepts in UMLS.

### Semantic filter construction

The results of the semantic filters are shown in Supplementary Table S4. Each semantic type involves at least one of the Reserve, Remove, or Error filters. A total of 33 432 entities were filtered. The Remove, Error, and Reserve filters, respectively, contained 2684, 3847, and 276 entities. In the table, ‘null’ indicates that no entities in that filter require processing.

### Semantic filter evaluation

**Figure 2.**
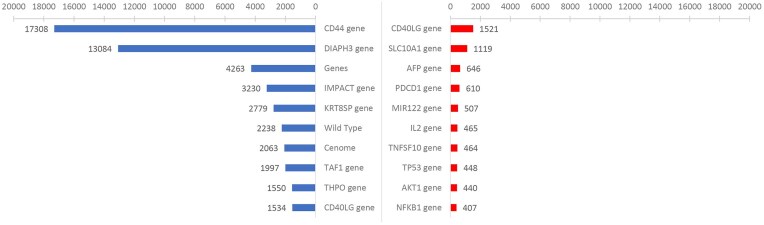
Top 10 entities by word count before and after applying semantic filters. Results of semantic filtering for the ‘Gene_or_Genome’ semantic in HBV. The left image shows the top 10 entities by word count before applying semantic filters, and the right image shows the results after applying the filters.

Figure 2 shows the results for the top 10 gene entities before and after applying filters using the ‘Gene_or_Genome’ semantic category in HBV as an example. The left and right panels represent the top 10 gene entities before and after filtering, respectively. The left image shows recognition results from unfiltered data, which include entities such as ‘Genes’ and ‘Wild Type’ that have no specific biological significance, and incorrectly identified and normalized entities such as ‘CD44 gene’. In contrast, the top 10 entities by frequency in the right panel were not only significantly reduced, but also biologically meaningful. For example, the top-ranked CD40 ligand (CD40LG) gene in its soluble form sCD40L can activate B cells to present the HBcAg18-27 peptide and act as antigen-presenting cells to induce HBV-specific cytotoxic T lymphocytes [25]. Similarly, the second-ranked SLC10A1 gene, with its Ser267Phe (S267F) variant, inhibits HBV infection [26]. This indicates the significant effect of the semantic filter in improving the accuracy of entity recognition.

Further evaluation (Supplementary Table S5) indicated that the MetaMap+ filter eliminated false positives identified by MetaMap. This resulted in 58% and 37% improvements in precision and *F*1 scores, respectively, compared with MetaMap alone.

### Analysing the importance of carcinogenic factors

**Figure 3.**
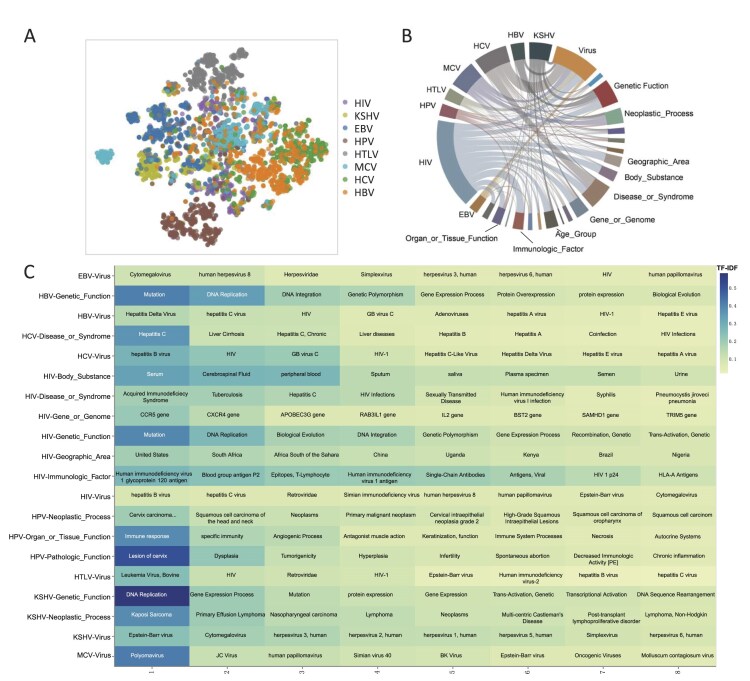
Analysis of the importance of viral carcinogenic factors. (A) The t-SNE plot of TF–IDF for eight types of viruses. Each point represents a document. A higher overlap of points from different viruses indicates greater similarity between the documents of those viruses. (B) Co-occurrence degree between virus terms and semantics. The co-occurrence degree is computed by taking the log of the *P*-value from the Fisher test. If there is a connection between a virus and a semantic term, it indicates a *P* < .05. In the plot, a larger segment area indicates a higher degree of co-occurrence. (C) Top 8 entities, sorted by TF–IDF, that have high co-occurrence with virus terms. The deeper the color, the higher the TF–IDF value.

The t-SNE plot (Fig. 3A) of TF–IDF revealed both specificity and similarity in the literature for the eight viruses. The text similarity of the relationships between HBV and HCV, HIV and HTLV, HPV and MCV, and EBV and KSHV was notably higher, whereas that of HPV and HTLV was the lowest.

Analysis of the co-occurrence of virus terms with different semantic types (Fig. 3B) indicated that seven viruses had high co-occurrence with the ‘Virus’ semantic type, whereas HPV did not. This suggests that there might be some co-infection among these viruses. The TF–IDF calculation results (Fig. 3C) further highlight that ‘Cytomegalovirus’ (EBV), ‘Hepatitis Delta Virus’ (HBV), ‘Hepatitis B Virus’ (HCV/HIV), ‘Leukemia Virus, Bovine’ (HTLV), ‘Epstein–Barr Virus’ (KSHV), and ‘Polyomavirus’ (MCV) are the most critical in their respective virus literature.

The HPV was most closely associated with the ‘Organ_or_Tissue_Function’ semantic, particularly in ‘Immune Response’, ‘Specific Immunity’, and ‘Angiogenic Process’, which had the highest TF–IDF values. KSHV showed high co-occurrence with ‘Genetic_Function’ and ‘Neoplastic_Process’, with the highest TF–IDF values for ‘DNA Replication’ and ‘Kaposi Sarcoma’. EBV exhibited high co-occurrence with multiple semantic types, including ‘Genetic_Function’, ‘Gene_or_Genome’, ‘Disease_or_Syndrome’, ‘Body_Substance’, and ‘Geographic_Area’. HCV was highly associated with ‘Disease_or_Syndrome’, with the highest TF–IDF values for ‘Liver Cirrhosis’ and ‘Hepatitis C’. HBV showed strong co-occurrence with ‘Genetic_Function’, particularly with ‘Mutation’ and ‘DNA Replication’, which had the highest TF–IDF values.

### Omics data analysis

**Figure 4.**
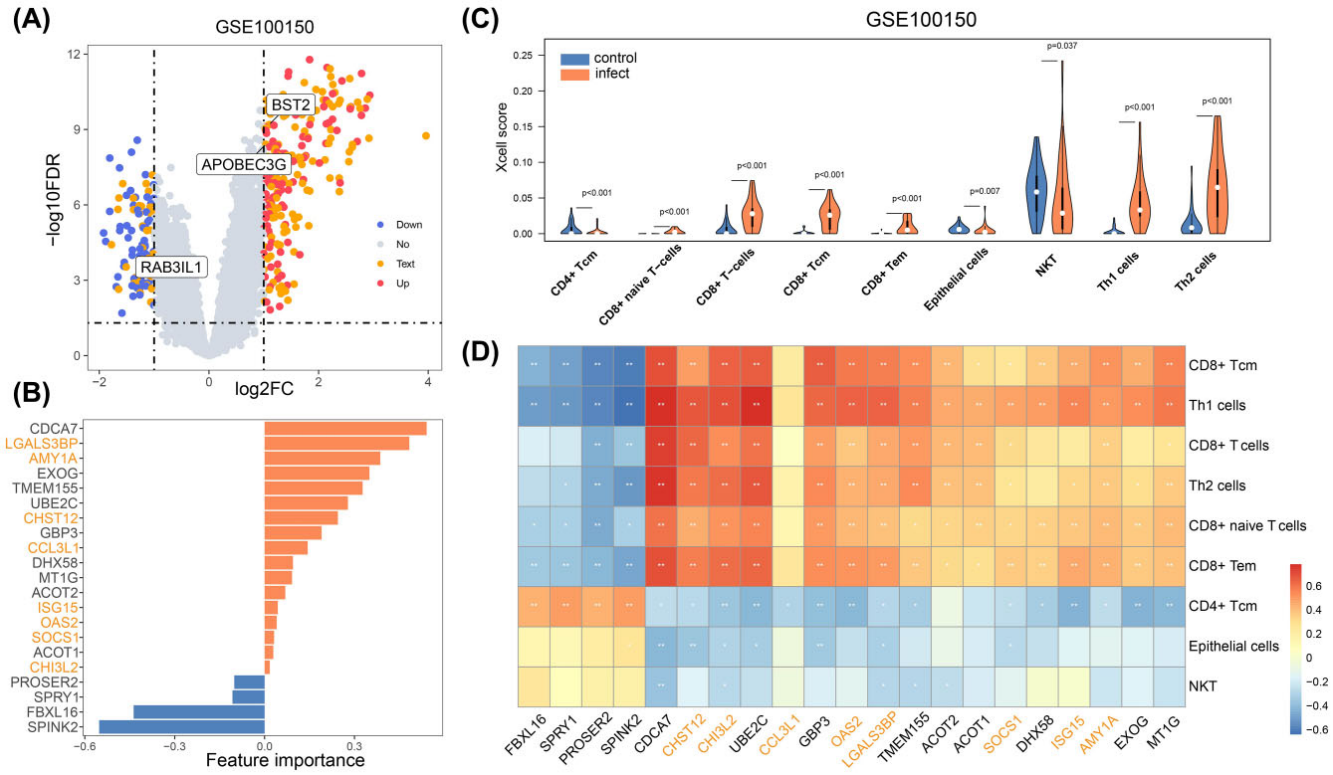
Omics analysis of GSE100150 dataset for HIV. (A) Gene expression differential analysis between HIV-infected and uninfected samples. (B) Feature genes selected through logistic regression, with genes marked in orange indicating those also identified in text mining. (C) Analysis of lymphocyte infiltration levels in HIV-infected and uninfected samples using the xCell tool. (D) Correlation analysis between feature genes and significantly differentially infiltrated lymphocytes.

We collected 47 sets of virus-related omics data (EBV, 15; HTLV1, 9; HIV, 7; HCV, 6; KSHV, 6; HBV, 2; HPV, 2). No data were collected for MCV. Each sample was categorized as either infected or uninfected, and further analysis was performed to compare differences in gene expression and immune microenvironment. For instance, the HIV GSE100150 dataset, which includes 63 samples, contains 28 HIV-infected samples and 35 control samples. Fifty percent of the DEGs were also identified through text mining. These genes include high-frequency genes in the text, such as APOBEC3G, RAB3IL1, and BST2. However, other high-frequency genes in the text, such as CCR5 and CXCR4, did not show significant differential expression in the omics analysis (Fig. 4A).

Through logistic regression analysis, we identified 21 key genes, of which 8 genes also appeared in text mining. Among these genes, CDCA7, SPINK2, and LGALS3BP exhibited the strongest feature importance (Fig. 4B). The immune microenvironment analysis focused on lymphocytes, revealing significant differences in their infiltration levels between HIV-infected and uninfected samples (*P* < .05). We observed that the infiltration levels of NKT cells, epithelial cells, and CD4^+^ Tcm cells were reduced in infected samples, while the infiltration levels of other T-cell subsets, including Th1, Th2, CD8^+^ naive T, CD8^+^ Tcm, and CD8^+^ Tem cells, were increased (Fig. 4C). In addition, we analysed the correlation between the feature genes identified by logistic regression and cell infiltration (Fig. 4D). We found that genes with positive values were negatively correlated with cells showing reduced infiltration and positively correlated with cells showing increased infiltration (e.g. CDCA7 and LGALS3BP), whereas genes with negative values displayed the opposite trend (e.g. SPINK2).

### Platform development

**Figure 5.**
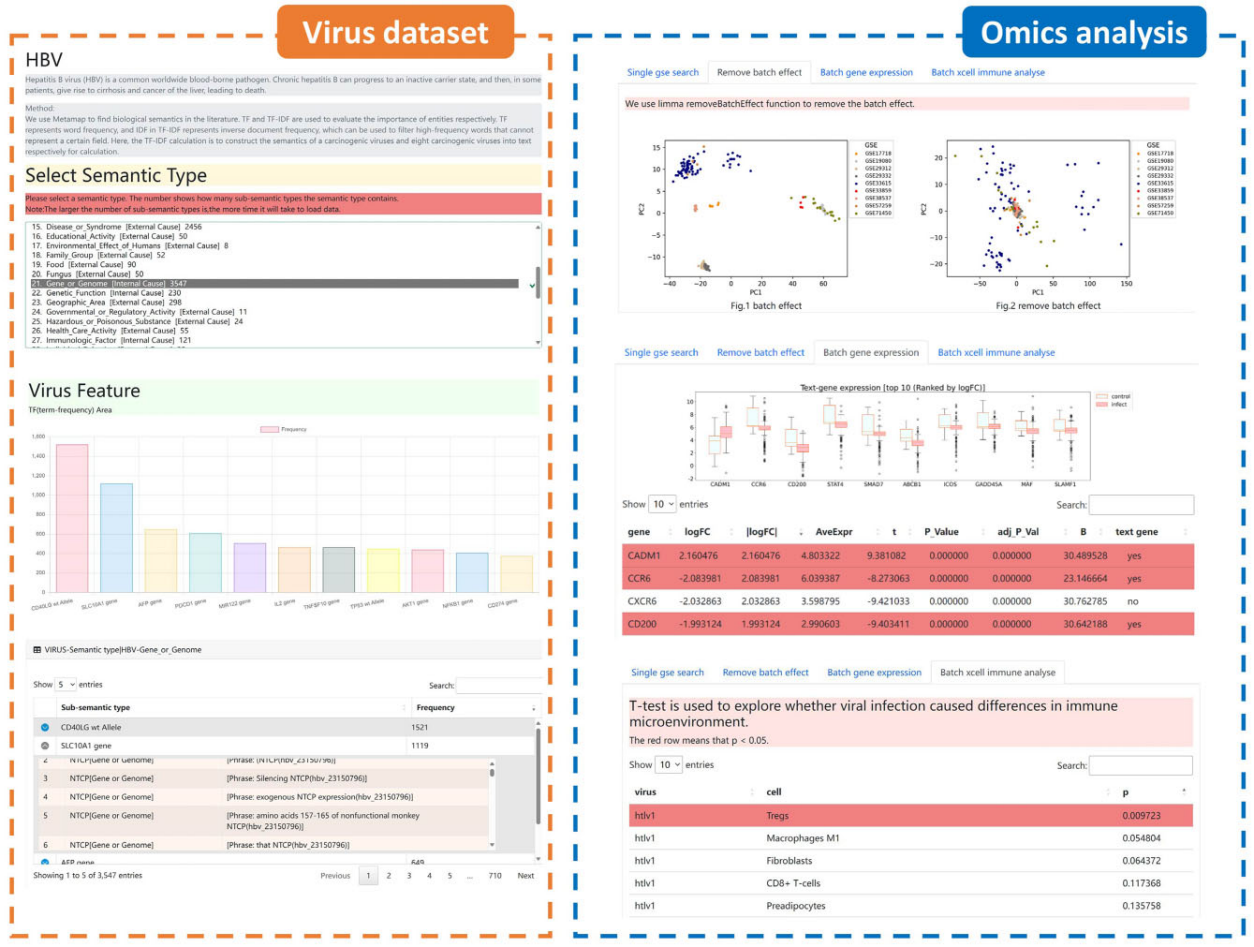
Platform development for viral literature annotation and omics data. The viral literature annotation dataset module includes TF–IDF and word count features for 43 different semantics. The omics data module includes the results of batch effect removal, gene differential expression analysis, and immune microenvironment analysis.

We developed a visualization platform—a virus dataset and omics analysis modules (Fig. 5). The virus dataset module includes TF–IDF and word count weights for entities under the 43 semantic categories for the eight types of carcinogenic viruses. This module was designed to extract key carcinogenic factors related to viruses from text data. The omics analysis module includes DEGs, feature gene selection, and immune microenvironment analyses. We also developed an optimized viral multi-semantic annotation tool that helps investigators to quickly extract key information from the literature in their fields of interest. The results and codes for the viral dataset and omics analysis are available for query and download at http://www.biomedinfo.cn:8281/.

## Discussion

This study explored factors contributing to cancer caused by carcinogenic viruses using text mining and omics analysis.

We developed a virus annotation dataset to identify key carcinogenic factors associated with viruses. By collecting literature on carcinogenic viruses, we recognized and normalized biomedical entities from the texts. We then built semantic filters to improve the accuracy of entity recognition, resulting in a comprehensive multi-semantic viral dataset. Analyses of word counts and TF–IDF features revealed similarities and specificities among eight viruses, with different semantic categories showing varying levels of importance for each. This might help to uncover potential key factors linking carcinogenic viruses to cancer development and provide valuable insights for investigators. However, virus annotation datasets have limitations. They primarily rely on the MetaMap tool for biomedical entity extraction, which requires improvements in recognition accuracy. Given the variety of entity recognition tools, we plan to explore other options such as PubTator [27] in the future to identify genes and diseases, and ViMRT [28] to detect viral mutations, that will enhance the overall effectiveness of viral text annotation.

We investigated the impact of carcinogenic viruses on gene expression and the immune microenvironment using omics data. We collected and annotated the omics data, and then analysed DEGs. We removed batch effects and integrated the data for a comprehensive analysis of altered gene expression to better understand the effects of carcinogenic viruses. We analysed the immune microenvironment using the xCell tool. Our results offer insights that can help investigators to mitigate the impact of carcinogenic viruses on cancer development by targeting gene expression regulation and immune response modulation. While this study primarily used microarray data for analysis, future research will incorporate various sequencing types, such as single-cell data, to explore the effects of carcinogenic viruses from multiple perspectives.

By integrating text mining and omics analysis, some overlapping genes were identified. These genes are not only are frequently mentioned in the literature but also exhibit significant differential expression or characteristic features distinguishing infected from uninfected samples in omics data. For example, the APOBEC3G gene encodes the A3G protein, which can be bound by HIV-1’s Vif protein, leading to its ubiquitination and degradation. This process eliminates the antiviral activity of A3G and promotes the successful replication of the virus [29]. The HIV accessory proteins Vpu or Nef can counteract the antiviral function of the BST2 gene through different mechanisms [30]. On the other hand, among the feature genes selected through logistic regression analysis, there were also genes that overlapped with the results of text mining, and these genes showed significant correlations with multiple T-cell subpopulations. For instance, the LGALS3BP gene has been confirmed to inhibit HIV infectivity. The discovery of these overlapping genes not only strengthens the reliability of the text-mining results but also further supports their critical role in HIV infection. However, some key genes identified through text mining did not exhibit significant expression differences. For example, genes CCR5 and CXCR4, identified via text mining as closely related to HIV infection and widely reported in the literature as co-receptors for HIV entry into host cells [31], did not show significant differential expression in omics data. This discrepancy may stem from differences in experimental conditions, such as sample origin, experimental design, or data-processing methods. This phenomenon also suggests that integrating text mining with omics analysis can be complementary, helping to reduce biases that may arise from a single data source and thereby providing a more comprehensive understanding of gene functions and regulatory mechanisms.

In summary, we constructed a multi-semantic virus annotation dataset, developed semantic filters to enhance entity recognition accuracy, and integrated histological data to identify key factors in viral carcinogenesis. Our findings revealed potential interactions among different viruses and provide a new theoretical foundation for the prevention and treatment of virus-related cancers.

## Supplementary Material

baaf038_Supplemental_Files

## Data Availability

The multi-semantic annotation dataset is available at http://www.biomedinfo.cn:8281/. The code can be used to annotate various biological semantic entity types from literature, and it further enhances the accuracy of entity recognition through our developed semantic filtering tool. The associated code repository is available at https://github.com/huanghonglian/multi-semantic-filter.
